# Collective reflections on the first cycle of a collaborative learning platform to strengthen rural primary healthcare in Mpumalanga, South Africa

**DOI:** 10.1186/s12961-021-00716-y

**Published:** 2021-04-19

**Authors:** Maria van der Merwe, Lucia D’Ambruoso, Sophie Witter, Rhian Twine, Denny Mabetha, Jennifer Hove, Peter Byass, Stephen Tollman, Kathleen Kahn, the Verbal Autopsy with Participatory Action Research (VAPAR)/Wits/Mpumalanga Department of Health Learning Platform

**Affiliations:** 1 White River, South Africa; 2 Centre for Global Development, School of Education, University of Aberdeen, Aberdeen, Scotland; 3 Aberdeen Centre for Health Data Science, Institute of Applied Health Sciences, School of Medicine, Medical Sciences and Nutrition, University of Aberdeen, Aberdeen, Scotland; 4 Department of Epidemiology and Global Health, Umeå University, Umeå, Sweden; 5 MRC/Wits Rural Public Health and Health Transitions Research Unit (Agincourt), School of Public Health, University of the Witwatersrand, Johannesburg, South Africa; 6 National Health Service (NHS) Grampian, Aberdeen, Scotland; 7 Institute for Global Health and Development, Queen Margaret University, Edinburgh, Scotland; 8 INDEPTH Network, Accra, Ghana

**Keywords:** Community participation, Embedded research, Collaborative learning platform, Primary healthcare, South Africa

## Abstract

**Background:**

Frontline managers and health service providers are constrained in many contexts from responding to community priorities due to organizational cultures focused on centrally defined outputs and targets. This paper presents an evaluation of the Verbal Autopsy with Participatory Action Research (VAPAR) programme—a collaborative learning platform embedded in the local health system in Mpumalanga, South Africa—for strengthening of rural primary healthcare (PHC) systems. The programme aims to address exclusion from access to health services by generating and acting on research evidence of practical, local relevance.

**Methods:**

Drawing on existing links in the provincial and national health systems and applying rapid, participatory evaluation techniques, we evaluated the first action-learning cycle of the VAPAR programme (2017–19). We collected data in three phases: (1) 10 individual interviews with programme stakeholders, including from government departments and parastatals, nongovernmental organizations and local communities; (2) an evaluative/exploratory workshop with provincial and district Department of Health managers; and (3) feedback and discussion of findings during an interactive workshop with national child health experts.

**Results:**

Individual programme stakeholders described early outcomes relating to effective research and stakeholder engagement, and organization and delivery of services, with potential further contributions to the establishment of an evidence base for local policy and planning, and improved health outcomes. These outcomes were verified with provincial managers. Provincial and national stakeholders identified the potential for VAPAR to support engagement between communities and health authorities for collective planning and implementation of services. Provincial stakeholders proposed that this could be achieved through a two-way integration, with VAPAR stakeholders participating in routine health planning and review activities and frontline health officials being involved in the VAPAR process. Findings were collated into a revised theory of change.

**Conclusions:**

The VAPAR learning platform was regarded as a feasible, acceptable and relevant approach to facilitate cooperative learning and community participation in health systems. The evaluation provides support for a collaborative learning platform within routine health system processes and contributes to the limited evaluative evidence base on embedded health systems research.

## Background

Community participation in health service planning and implementation is known to generate significant benefits to the health and healthcare of all people, including improved access to care, improved health and clinical outcomes, disease prevention, better health literacy and self-care, and reduced overall costs [1–5]. The rights and duties of communities to individually and collectively participate in the planning and implementation of healthcare have been acknowledged as a founding principle of primary healthcare (PHC) over the past four decades [6]. More recently, the 2030 Agenda for Sustainable Development recognizes that health goes beyond survival and includes human rights, equity and the empowerment of vulnerable populations [7].

The need for participatory governance and accountability, bringing in the voice of end users of health services and empowering them to participate in public decision-making, is increasingly recognized as a requirement for the effective implementation of health policies and strategies [8]. The evidence base on potential mechanisms for governments to engage with populations, civil society and communities is growing, with a best-practice guidance document currently being developed by WHO [9, 10]. It is however not clear whether and how participatory research, as a health systems strengthening initiative, can be embedded within the system and contribute toward the enhancement of community participation in health system processes and decision-making.

In the health field, community participation is mostly regarded as a process to support health programmes to sustain health improvement outcomes, rather than an intervention per se [11]. Despite global and local acknowledgement of the critical role of community participation in health service organization and delivery, community participation is mostly restricted to health promotion interventions and rarely applies to governance processes [12]. The health policy and planning processes seldom allow space for participation.

The current progressive and inclusive post-apartheid legislative and policy context in South Africa provides for community participation to address past inequalities through people-centred services. In the health sector, this includes an express commitment to the right to health and to community participation in PHC [13]. The district health system is the organizational unit for health service implementation, bringing health services closer to communities. However, frontline managers and providers are often constrained from responding to community priorities due to organizational cultures characterized by supervision and management systems focused on compliance with centrally defined outputs and targets [14, 15].

Roles for different spheres of government are defined in policy documents, but mechanisms for stakeholder participation that would enable the learning and feedback towards successful implementation of health strategies and interventions are lacking [16]. Community health workers (CHWs) are regarded as the interface between community systems and the health system. South Africa adopted a ward-based primary healthcare outreach team (WBPHCOT) strategy in 2011 as part of PHC re-engineering. A five-year policy framework and strategy for WBPHCOT was established in 2018, with community participation and empowerment as key principles [17]. While this cadre of health workers are responsible for community engagement, they are often severely constrained in terms of capacity and work environments [17–20].

Facilities at the frontline of service delivery have strongly voiced a requirement for local, disaggregated information and experiential knowledge to make locally appropriate and responsive decisions and to allow them to incorporate policy interventions into everyday routines and practices [21]. The large volumes of data produced by health management information systems are used minimally for local health decision-making, constraining responses to local priorities [22]. National health indicators are primarily based on centrally valued information from the District Health Information System (DHIS) and are utilized during planning and implementation processes such as target setting, activity planning and budgeting. This is often perceived by health managers as a disincentive to plan based on community priorities [4, 14]. In addition, the epidemiological approach towards health system planning is mostly based on disease priorities and does not consider local lived experiences of service users, barriers to health at the community and health system levels or the social determinants of health [4, 23].

**Fig. 1:**
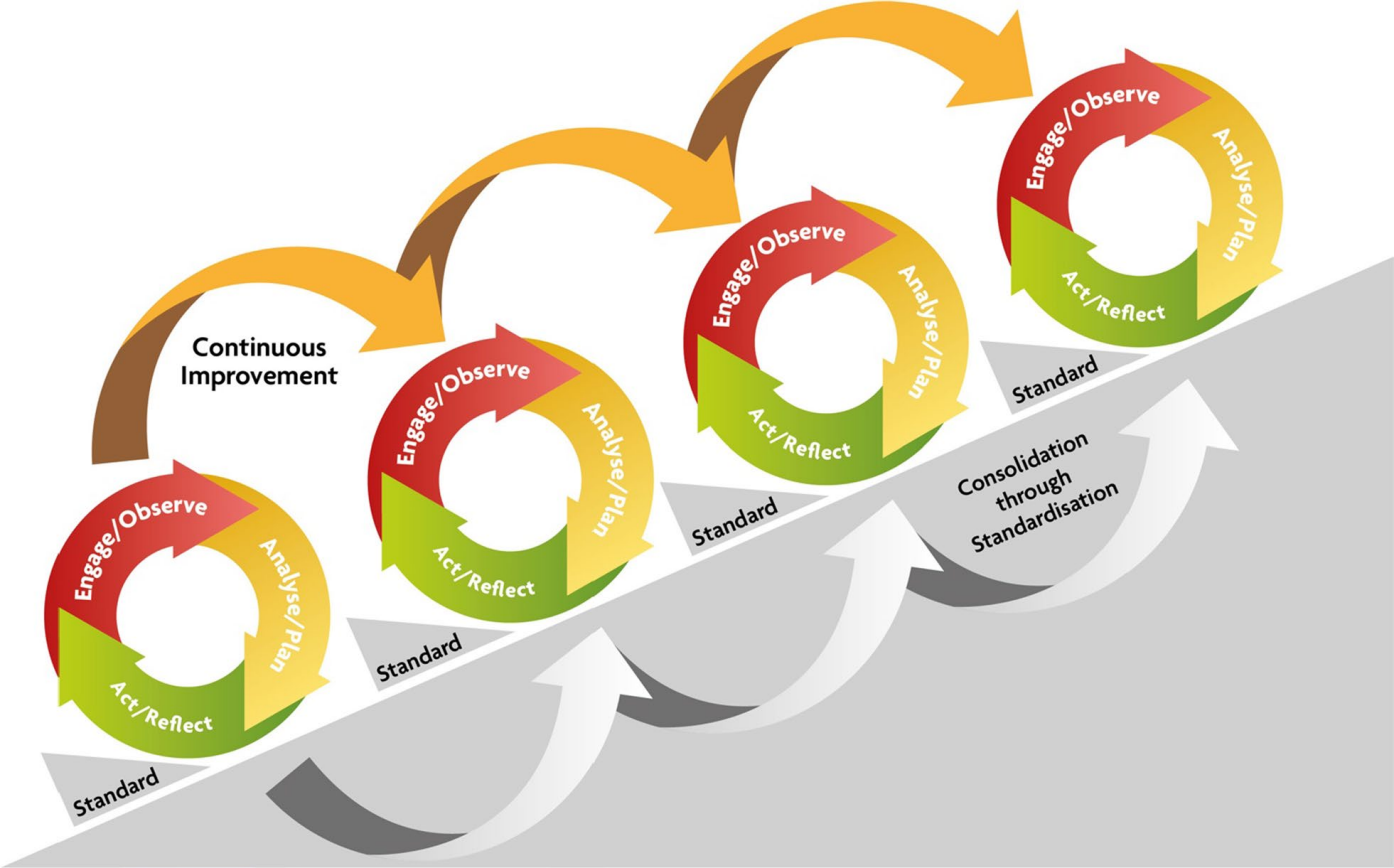
Design and time frame of the VAPAR programme

The Verbal Autopsy with Participatory Action Research (VAPAR) programme [24] is a collaborative learning platform embedded in the health system in a rural province in South Africa, with a commitment to building partnerships between communities, health authorities and researchers through a series of action-learning cycles (Fig. 1). During the first cycle, two challenges were identified as priorities by community and health system stakeholders, namely lack of clean safe water and its links to under-five mortality, and alcohol and other drug abuse, linked to adolescent health. The process through which these health priorities were collectively identified with health authorities and community representatives has been described elsewhere [25–27].

This paper presents the results of collective reflections between researchers, communities and authorities, through an evaluation of one round of the VAPAR learning platform. The aim is to inform its development and, more broadly, drive understanding of the potential of and modalities for embedded and participatory health systems research as a health system strengthening initiative. The objectives were to conduct a theory-informed evaluation of the first action-learning cycle (August 2017 to November 2018) in the VAPAR programme, collectively reflect on the process with communities and authorities, gain insights and learning for subsequent cycles, and identify opportunities for integration with health system processes, as well as future linkages and collaboration. This study furthermore provided a first stage of and formative basis for the overall participatory evaluation of the VAPAR programme, through a revision of the initial programme theory of change [28].

## Methods

### Theoretical argument

The evaluation drew on existing links in the provincial and national health systems, informed by the health policy and systems research paradigm. Subscribing to enquiry paradigms asserting that reality is multiple, relative and socially constructed, this emerging field brings social science perspectives to bear on key health system and development issues [29, 30]. On this foundation, a pragmatic and mixed-method approach was applied.

Prior to the evaluation, the researchers developed the initial programme theory through a process of reflective exchange and drawing on existing literature as well as their experiences and insights from pilot work in 2015–16 [28]. The programme theory of change articulates the complex interplays between context, mechanisms of change and outcomes. Realism applies the concept of mechanism to understand the relationship between context and outcome—to build insights into what it is about programmes and interventions that bring about effects, for whom, to what extent, under what circumstances and why [31]—and was consequently considered as an appropriate evaluation approach for the programme. Realist methodology can be customized to the needs of participatory research assessment and offers an opportunity to advance theoretical understanding of the processes and contexts of implementation that yield impacts, through refinement of the initial programme theory, by means of a context-mechanism-outcome configuration [32].

### Setting

South Africa is a medium-sized country with a culturally diverse population estimated at 58.8 million [33]. Eight decades of structural discrimination in favour of a minority population group during apartheid formally ended with the nation’s first democratic election in 1994. Health and economic disparities however remain and are further aggravated by slowing local and global economic prospects in recent years, with resulting persistent high poverty, inequality and unemployment [34–36]. The country is furthermore experiencing a rapid epidemiological transition, with a 26.8% probability of death due to noncommunicable disease before the age of 70 years, while the communicable disease burden remains substantial, and an estimated 7.97 million people are living with HIV [33, 37, 38]. In addition, the overall disease burden remains higher in lower socioeconomic groups due to entrenched structural inequalities [39]. This persistent and widening disproportionate burden of avoidable morbidity and mortality is aggravated by poor access to PHC for poor and rural communities [35, 40].

The research was conducted in Mpumalanga, a rural province in the northeast of South Africa. Mpumalanga is one of nine provinces in the country, with a population of almost 4.6 million, 7.8% of the national population [33]. The VAPAR programme is grounded in a collaboration with the provincial health authority and based at the MRC [Medical Research Council]/Wits Rural Public Health and Health Transitions Research Unit (Agincourt), which hosts the Agincourt health and sociodemographic surveillance system (HDSS) [41]. Established in 1992, the Agincourt HDSS is the longest-running HDSS in South Africa, generating longitudinal data on vital events (deaths, births, migrations) for a population of approximately 120,000 individuals from 31 villages [42]. About a third of the population in the HDSS site are migrants from Mozambique, and socioeconomic and health conditions in the area are characterized by limited piped water and basic sanitation, underdeveloped roads, and high unemployment amidst a high HIV/AIDS and rapidly increasing noncommunicable disease burden [42–44].

The VAPAR programme was co-designed with the provincial Directorate for Maternal, Child, Women and Youth Health and Nutrition (MCWYHN), with continued representation of this directorate as co-investigators in this programme. The programme consists of a series of action-learning cycles to progress intersectoral engagement and to confer power to community stakeholders (Fig. 1). The design of the programme allows for continuous engagement with health officials at different levels and from different sections in the health system, as well as other relevant stakeholders.

### Design

The study involved three data collection phases: individual discussions with VAPAR programme stakeholders over 2 months (April and May 2019); an interactive workshop with provincial Department of Health (DOH) managers (May 2019); and a workshop with national child health experts (May 2019). Findings from the three phases were reviewed, collated and fed into a revised programme theory. The evaluation focused on specific layers of outcomes from the framework of the initial programme theory in order to systematically identify the context-mechanism-outcome configurations that drive these outcomes. These outcomes included research and stakeholder engagement, organization and delivery of services, establishing an evidence base for policy and planning, and improving health behaviours and outcomes .

### Data collection

#### Phase 1: Stakeholder discussions

This phase involved individual face-to-face discussions with 10 stakeholders who had participated in the first cycle of the VAPAR programme. Stakeholders represented the different constituencies in the learning platform. Individual participants were identified through maximum variation sampling and recruited telephonically by the researchers, with a date and venue for individual meetings arranged as suitable for each participant. The participants included five officials from government departments and parastatals, as public entities; two representatives from nongovernmental organizations (NGOs); and three representatives of the local communities involved in the VAPAR programme. The purpose of the individual discussions was to capture personal experiences of and reflections on the programme mechanisms of change, contextual construct and early outcomes. Discussions were facilitated by a VAPAR co-investigator familiar to the participants, with background experience as program manager in the public sector. Informal individual discussions were guided by a discussion framework and focused on the four outcome  highlighted above. The facilitator made notes of the key discussion points during the discussions, and the discussions were not recorded.

#### Phase 2: Provincial workshop

Building on the individual discussions, the second phase involved an interactive workshop with nine managers in the provincial DOH, from programmes and directorates relevant to the health challenges identified by the communities during the pilot phase and first cycle of the programme, including PHC, MCWYHN, HIV/AIDS and tuberculosis, and community-based services. An invitation to the workshop was forwarded to the health authority, with an indication of the relevant programmes, and the final attendees were selected by the health authority. The workshop was facilitated by the researchers and guided by a semi-structured agenda, to allow for discussions to be focused but flexible according to participants’ inputs. Findings from the individual stakeholder discussions (described above) were presented to the workshop participants, along with an initial consideration by the researchers on mechanisms to integrate the learning platform into routine health system processes. This was followed by a discussion on the contexts and mechanisms of change and the outcomes, as reflected in the theory of change framework, of the first cycle and insights for the planning of future programme cycles, as well as the levels and mechanisms for integration of the VAPAR programme into the provincial health system. The levels and functions for integration of the VAPAR programme into the provincial health system was captured and displayed electronically during the provincial workshop, allowing for further deliberation and consensus-building. A report of the workshop was shared with participants for validation.

#### Phase 3: National workshop

In the third phase, we engaged with five child health experts drawn from the Ministerial Committee on Morbidity and Mortality in Children under five (CoMMiC) from four provinces, as health programme and policy specialists relevant to the health-related challenges identified by community and health system stakeholders. Invitations to the workshop were forwarded to the child health experts individually, and the workshop was arranged at a date and venue convenient for the participants. An interactive workshop was facilitated by the researchers and guided by a semi-structured agenda to allow for contributions and discussions by the participants. An overview of the VAPAR programme was presented, with a focus on the verbal autopsy (VA) innovations regarding the causes and circumstances of avoidable mortality in children under 5 years of age, as well as insights from the participatory action research (PAR) process on how these could be addressed collaboratively. Discussions during the workshop focused on health system research processes and the identification of gaps and opportunities to embed such processes into the health system. A report of the workshop was shared with participants for validation.

### Data management and analysis

Notes taken by the researchers on the content of and interaction during the individual stakeholder discussions were summarized and a basic descriptive thematic analysis done for each stakeholder constituency (government, NGO and community representatives), exploring the contexts, mechanisms of change and outcomes as described above. The data were further thematically coded and analysed to identify commonalities and divergences between the stakeholder categories. Data sources, including workshop presentations, notes and outputs, as well as internal programme documents (visual data, social media posts, programme briefs and reports) and data were collated and reviewed by the researchers according to the four identified outcomes prioritized in this evaluation. Following data analysis, the researchers reviewed and revised the initial programme theory of change to reflect stakeholder perspectives as lived experiences from health service providers and users.

## Results

### Individual discussions

Programme outcomes described by stakeholders participating in the individual discussions are summarized in Table 1. In the contexts of deep divisions, lack of trust and dialogue between services and communities, stakeholders considered the cooperative and participatory approach in the VAPAR programme to have had an impact on research and stakeholder engagement, and encouraged continued engagement between stakeholders. Government-based stakeholders perceived engagement to sometimes be limited to the VAPAR process, while parastatal and NGOs stakeholders reported pre-existing and ongoing engagement. Community stakeholders felt empowered through the process and reported having increased confidence to further engage with official bodies and structures, including the local municipality, to communicate the needs of the community.

**Table 1:** Outcomes described by VAPAR stakeholders

Level	Outcomes identified by interviewed stakeholders
Research and stakeholder engagement	Appropriate platform for the Department of Health to engage with community members, allowing collective identification of health-related challenges and planning to address these challenges Assisted with role clarification among different government departments, parastatals and NGOs, thereby identifying areas for collaboration towards specific goals, and opportunities to hold each other accountable for respective responsibilities Achieved engagement among stakeholders from different constituencies, including government and parastatals, nongovernmental organizations and community members Empowered community stakeholders to further engage with official structures
Organization and delivery of services	Improvement in the delivery of water to communities in the study site (as a priority area identified during the first VAPAR cycle) recognized and acknowledged as a perceived programme outcome by community-based interviewees Delivery and organization of health services in general and specific to children under 5 years of age (as a priority identified during the VAPAR pilot phase) not regarded to have notably improved during this period Improvements in law enforcement with regard to the trading hours of taverns, as well as noise levels, were reported by one of the community-based interviewees and attributed to the VAPAR process through which senior police officials became aware of the community concerns
Establishing an evidence base for policy and planning	Potential to influence policy and planning generally acknowledged Community engagement, consultation and participation could lead to improved policy and planning
Improving health outcomes	Community awareness, education and engagement regarded as ways to improve health behaviour and therefore also health outcomes over time No direct improvement in health outcomes demonstrated to date

VAPAR stakeholders included officials from government departments and parastatals, representatives from nongovernmental organizations and local community representatives who had participated in the pilot phase and first cycle of the VAPAR programme

Individual stakeholders also described outcomes with regard to the organization and delivery of services, and mostly agreed that the VAPAR process has potential for service delivery improvements, as it is informed by the reality on the ground, and promotes collaboration and accountability among stakeholders. Service delivery improvements attributed to the VAPAR programme are cautiously interpreted due to the political timing of the discussion (in a period leading up to a national election, with expected accelerated efforts to improve service delivery). Notwithstanding the cautious interpretation, there was a generally positive view among government stakeholders that service delivery improvements could be realized if government structures collectively act on the needs and priorities of the community and address these jointly with community structures.

The potential for impact on policy and planning was generally acknowledged. In the view of the individual stakeholders, community engagement, consultation and participation would lead to improved policy and planning, as community needs would be prioritized, rather than policy decisions being based on selective or biased evidence and political priorities. It was anticipated that communities would buy into the policies and plans if they had been involved in the process. At the same time, governmental stakeholders were mindful of financial and resource limitations within the public sector, which impact service delivery and could limit the integration of VAPAR programme processes into routine government processes.

Although no direct improvement in health outcomes had been demonstrated, community awareness, education and engagement were regarded as ways to improve health behaviour and therefore also health outcomes over time. The VAPAR process was considered acceptable, relevant, participatory, inclusive of “community voice”, non-prescriptive and owned by stakeholders. The stakeholders expressed optimism for integration of the VAPAR process into routine services, although with concerns from government stakeholders on the potential adverse effect of budgetary and other resource constraints.

### Provincial workshop

It was noted that there was potential for VAPAR data and processes to be incorporated into routine health system planning, for officials from the department to participate in all stages of VAPAR and for the VAPAR process to support community participation in routine health system processes (Table 2). It was also recognized that data generated through the VAPAR programme provide insights into the lived experiences of communities and could supplement information relating to morbidity and mortality that are generated through clinical assessment processes.

**Table 2:** Areas of suggested integration of VAPAR into routine provincial health systems in Mpumalanga Province

Level	Function	Integration
Provincial	Annual performance plan	Implied through collaborative district involvement, through a “bottom-up” approach
District	District health plan	Attend district health management team meetings, to present data and process. Participate in development of the district health plan
Subdistrict	PHC management	VAPAR to have a slot in the quarterly subdistrict PHC meeting to present programme to all PHC and operational managers and shared learning from Agincourt “pilot” facilities
PHC facility	Operational management	Skills exchange and capacity-building with operational managers. Operational managers to participate in VAPAR with a focus on analysing, planning and acting on community evidence into service organization and delivery
Community	Outreach teams	Skills exchange and capacity-building with CHWs. CHWs to participate in VAPAR, with a focus on the community engagement element

On presentation and review of VA data on deaths in children under five, a general assumption by health workers about the use of traditional medicine at and around the time of death was challenged, with VA data on critical, limiting circumstances of medical outcomes not supporting these assumptions. The discussions recognized the potential of these data, when co-produced, owned and used routinely for local level everyday decision-making, to challenge assumptions and inform decisions over resource allocation.

Workshop participants appreciated the multisectoral and multilevel design towards addressing social determinants of health as identified by community participants, with recognition that the mandate to address many of these determinants does not lie with the DOH. Evidence of programme outcomes from the individual discussions was verified during an interactive group discussion and the potential for the process to contribute to evidence-based planning and service organization acknowledged by workshop participants. It was proposed that this potential could be realized through integration of the programme with routine health system processes and through a skills exchange by inclusion of frontline health workers in the programme processes.

### National workshop

Child health programme and policy experts were receptive towards the programme design and outputs at the time of the evaluation, including data on circumstances of mortality and place of death derived from VA, as well as on social determinants and broader social and cultural norms highlighted through PAR. Some refinement of place of death and circumstances of mortality construct was recommended for the VA data.

Regarding the next cycles of the programme, these participants suggested that VAPAR could acquire insights on how existing auditing initiatives and processes within child health programmes, such as the Child Healthcare Problem Identification Program (ChIP) and *Operation Sukuma Sakhe*, perform useful functions and managed to scale up by being embedded into routine health functions (Table 3). The group advised that while mortality audits are generally done well for facility-based events, with good coverage and by different groups, the need for support and input around community participation is an area where useful contributions could be made.

**Table 3:** Existing initiatives within Maternal, Child, Women and Youth Health and Nutrition programmes in South Africa

Intervention	Aim/purpose	Description
Operation Sukuma Sakhe (KwaZulu Natal province)	Aims to integrate and coordinate the efforts of all stakeholders to improve the lives of communities The desired outcome of the service delivery model is the implementation of a comprehensive, efficient, effective, quality service delivery system that contributes to a self-reliant society in a sustainable manner Priorities: –Rural development/agrarian reform and food security –Creating decent work and economic growth –Fighting crime –Education –Health	Ward-based approach, prioritizing vulnerable households *Step 1* –Community caregivers (CCGs) visit a set number of households where a key informant (particularly the household head) provides information on individuals, household and community needs –The household profiling tool is completed by the CCGs and the baseline is identified –Youth ambassadors (YAs) meet with youth at households, schools, churches, clubs, etc., to jointly identify needs and challenges of youth *Step 2* –CCGs and YAs take the baseline information to the war room each week –War room members assess the needs, and priority (immediate) needs are identified –YAs work with youth to address the needs and challenges of youth *Step 3* –War room discusses the needs and submits information to referral focal point person in each department for action –Weekly baseline data are consolidated and submitted to the local task team and to the relevant departments for action –Departments provide services via the war room –CCGs provide feedback to households –At ward level, solutions are discussed with government and other partners to embrace youth programmes –YAs provide feedback to youth
Child Healthcare Problem Identification Programme (ChiP/Child PIP)	Mortality audit tool designed specifically for infants and children (from birth up to 18 years) The Child PIP programme aims to use the information gathered from careful mortality review to improve the quality of care sick children receive in the health system	*1. 24-h review* Every death summarized within 24 h by the on-duty intern/medical officer or registrar to obtain all necessary information *2. Preparatory meeting* Before mortality review meeting, attended by doctor and nurse in charge of ward, to conduct a detailed analysis of all deaths, select cases for presentation and compile monthly statistics *3. Mortality meeting* Held weekly to monthly. Attended by the whole paediatric team including PHC clinic staff, to present statistics and cases in order to identify, assign and review tasks *4. Epidemiology and analysis* Quarterly, six-monthly and annually. Attended by managers and clinical personnel for broader problem identification

### Synthesis

Collective recommendations for future action-learning cycles from the three data collection phases are summarized in Table 4.

**Table 4:** Recommendations for future VAPAR learning-and-action cycles

Recommended by	Recommendation
Government, NGO and community stakeholders participating in individual discussions	Include local municipal managers during all stages of prospective action-learning cycles
	Convene stakeholders at the end of each VAPAR cycle for collective reflection and learning
Provincial DOH workshop participants	PHC clinic operational managers and CHWs to be included at all stages of the next action-learning cycle of the programme, with a focus on skills exchange
	VAPAR representatives to participate in routine district and subdistrict planning and reporting processes, including development of the district health plan and quarterly performance review
	Alignment/integration of VAPAR programme into existing health structures at critical levels of engagement, primarily at household/community (CHW/ward-based primary healthcare outreach team) and subdistrict (clinic operational managers, PHC supervisors) level
	Focus on community participation and contemporary priorities—support strengthening the management model of PHC facility manager, and consider other programmatic priorities such as adolescent and mental health
National workshop participants	Refinement of VA with regard to place of death/circumstances of mortality construct
	Continued engagement with CoMMiC to report on progress and inform future development/application and feeding up into national learning

**Fig. 2:**
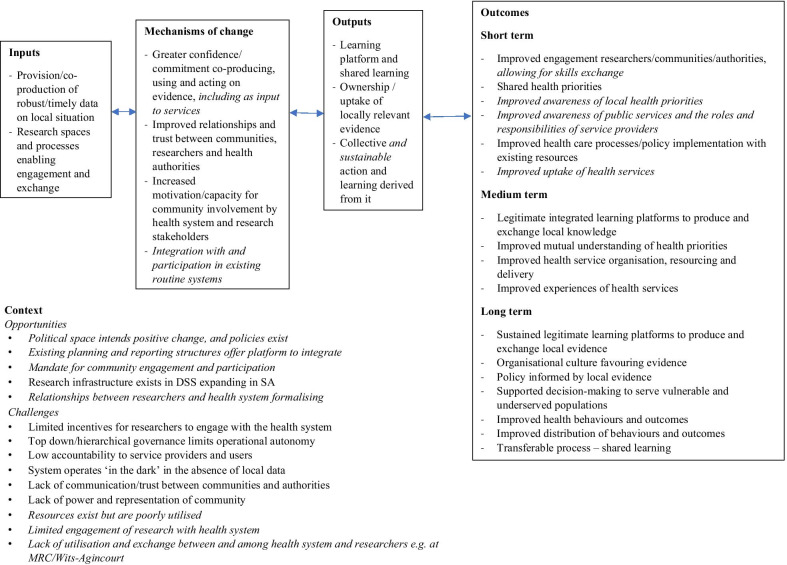
Revised VAPAR programme theory of change, with revisions indicated in italics

Following completion of the three elements of the evaluation, the researchers revised the initial programme theory of change [28] to synthesize the learning. The revised programme theory (Fig. 2) incorporates refined insights into the contexts, mechanisms and outcomes of the programme from stakeholder perspectives. We added elements on participation in routine health system planning and reporting processes by VAPAR team members, along with the inclusion of frontline PHC officials during all stages of the next programme cycle, as opportunities for skills exchange. Expected outcomes were updated to include improved understanding of public services as well as of the roles and responsibilities of different stakeholders, thereby improving service uptake by community members. The programme context was updated to reflect more nuanced understandings of challenges and opportunities within the rural PHC setting.

## Discussion

Recognizing the potential for health system strengthening through a collaborative learning platform, this paper aims to develop an understanding of the contexts, mechanisms of change and outcomes, as well as potential impacts, of a research programme embedded in the local health system, to identify opportunities for strengthening the VAPAR programme. Stakeholders were generally appreciative of the VAPAR programme in terms of the process and data generated. The programme was regarded as unique in design, through the participatory approach and extensive involvement of community representatives throughout the process.

While being at a relatively early stage (first of a series of action-learning cycles), the programme is providing encouraging evidence that communities are willing and able to contribute rich information on the social and health system dimensions of avoidable mortality, and that the process of doing so can potentially be used to connect communities, researchers and health authorities in evidence production and exchange as a basis to inform service delivery, policy and planning [26, 45, 46].

The findings of this evaluation provide relevant and context-specific information on the local health system in a rural province in South Africa and suggestions to improve community participation in the health system, from local and subdistrict levels upwards in the decentralized health system with centralized tendencies [21]. This evaluation identified the potential of the VAPAR programme to impact the organization and delivery of services at the operational level, as it provides a platform for government structures to collectively act on the needs and priorities of the community and address these jointly with community structures, applying local information and experiential knowledge for responsive decision-making and action.

Embedding of locally relevant and demand-driven research in health systems provides a means to improve the implementation and scale-up of health policies and contribute to the achievement of health-related goals [47, 48]. A further advantage is that an understanding of organizational culture allows for research to be contextually relevant, thereby potentially closing the evidence-to-policy gap by increasing ownership and improving the uptake of evidence-based strategies into routine system functions [48, 49].

In other low- and middle-income countries, decision-makers expressed overall satisfaction and positive perceptions of embedding research in health policy and systems decision-making [50]. This affirms that active engagement of policy-makers from the onset of and throughout the research process can address barriers to evidence uptake, namely engagement and ownership of end users and applicability of relevant and context-specific research. Nevertheless, in already overburdened health systems, time was identified as a barrier to embedding of research in health policy and planning [50]. This refers to the time required for research and time to bring about changes and to reach agreement among stakeholders. The VAPAR research team is aware that building consensus requires intensive engagement and time from researchers and decision-makers, grappling with various competing interests. Integration within the health system, rather than a parallel process, reduces the time and resource demands on busy staff.

Embedding the VAPAR programme into routine health system planning and review processes has the potential to elevate community participation in these processes and the recognition of health barriers and enablers identified by local communities, as demonstrated in other settings. Bringing together the “triangle that moves mountains” (government, communities and researchers), as demonstrated in the National Health Assembly in Thailand, allows for diverse stakeholders to contribute to participatory governance in health systems [51]. Similarly, an initiative aimed at community engagement in health system planning in Kenya had a positive effect on target setting and prioritization, although the overall planning process remained responsive to indicators chosen according to national priorities [4].

During this evaluation, CHWs were identified as a cadre to be directly involved during subsequent cycles of the VAPAR programme, with a particular focus on strengthening their community engagement skills. In addition to their scope of practice, the South African national policy framework for CHWs promotes the values of community participation and empowerment, as well as intersectoral collaboration [17]. However, the implementation context within which CHWs operate is constrained, and there is a call for CHW programs to be regarded as a full-fledged subsystem of the PHC and district health system, rather than just human resources [20]. Consideration should also be given to the multiple, systemic challenges and constraints experienced by CHWs. These include varying perceptions of their role in the health system, lack of financial support, poor governance, challenging work and operational environments, a lack of employment security, and inadequate supervision stakeholder and community support [18, 19].

Health-related priorities identified by community stakeholders in the VAPAR programme require intersectoral action. Collaboration between different constituencies has the potential to bring about improvement in the health status of people, but it is not always clear by whom and how such collaboration can be initiated or sustained [52, 53]. The VAPAR programme provides a platform for intersectoral collaboration, with stakeholders playing different roles towards a common purpose. In this evaluation, government-based stakeholders perceived engagement between stakeholders to sometimes be limited to the VAPAR process, while parastatal and NGO stakeholders reported pre-existing and ongoing engagement with other constituencies. Although a structured platform for intersectoral collaboration is helpful, competing priorities may limit collaboration towards a common purpose. While multisectoral collaboration is recognized as pivotal for the improvement of health outcomes, this has not been realized. Collaboration between constituencies is typically hampered by organizational contexts which include poor governance, lack of collaborative working, limited consideration for citizen voice and a lack of trust [54, 55].

Other formative research involving communities in the identification of health behaviours has illustrated that community groups were keen to act but were often unsure about what to do, and recommended capacity-building to enable participants to engage with policy-makers and health workers [56]. Community stakeholders in VAPAR however indicated that the programme has empowered them to engage with government and other structures at the local level, outside of the programme.

The design of the VAPAR programme addresses concerns that researchers working in the health policy and system field are not sufficiently engaged with real-world experience, taking daily realities of health service provision into account [57]. The triangle of researchers, community representatives and health system actors ensures relevant context-specific and realistic actions to address the health-related challenges identified through the programme. Being based in a HDSS furthermore improves access to health and demographic data, for health workers and community members, aiding in the identification of health challenges and behaviours and therefore the pinpointing of modifiable factors.

In relation to limitations, members of the research team led the individual discussions and workshops, which may introduce positive results bias. This risk was mitigated, but may not have been eliminated, through deliberation by the research team and peer review of this evaluation by an independent programme steering committee. In addition, the workshops were only attended by stakeholders from the health sector, and workshop discussions therefore do not reflect insights from other constituencies with regard to embedding the research process into routine health services. This paper acknowledges but does not focus on elements that underpin health service delivery, such as financing or human resource constraints, or the political and social forces that affect health system development and policy implementation [28]. Evaluation of a collaborative learning platform requires critical attention to issues of empowerment and ownership of health improvements, which have not been extensively addressed in this evaluation.

This evaluation reflects the collective views and inputs of researchers, health system stakeholders and community representatives, providing insights and learning from within the health and local community systems. The COVID-19 pandemic brought the importance of policy learning to the forefront, with inclusive and collaborative community responses demonstrated to be powerful in the context of crisis situations where responses through formal decision-making structures may be slow and limited [58, 59].

## Conclusions

This evaluation contributes to the limited evaluative evidence base on embedded and participatory system research as a health system-strengthening initiative, with a view to enriching global learning as well as enhancing this collaborative learning platform. Overall, a learning platform embedded within the local health system and integrated with routine planning and review processes is regarded as a novel and relevant approach for facilitating collaborative learning and community participation in health systems.

During all three phases of the evaluation, stakeholders were appreciative of the programme in terms of the data and process generated. Some promising outcomes were noted with regard to engagement between health system stakeholders as well as further potential for impact on health system organization, policy and planning, and ultimately health outcomes, through this collaborative learning platform.

The evaluation provides support for a collaborative learning platform within routine health system processes such as planning and review, thereby strengthening the research-practitioner linkages. The VAPAR platform is built on trust and collaboration between stakeholders, which is often lacking between communities and health systems, and which is increasingly important at a time of global pandemics and health emergencies. The findings of this evaluation generated important suggestions on how to adapt the VAPAR programme model during the next cycles, through a reciprocal agreement to integrate the programme with routine health system processes and inclusion of frontline health workers in the programme processes. The evaluation findings will also feed into the overall VAPAR programme evaluation, which is an integral part of the learning process.

## Data Availability

The datasets used and/or analysed during the current study are available from the corresponding author on reasonable request.

## Abbreviations

CCG: Community caregiver
ChIP: Child Healthcare Problem Identification Programme
CHW: Community health worker
CoMMiC: Ministerial Committee on Morbidity and Mortality in Children under five
DHIS: District Health Information System
DOH: Department of Health
HDSS: Health and sociodemographic surveillance system
MCWYHN: Maternal, child, women and youth health and nutrition
NGO: Nongovernmental organization
PAR: Participatory action research
PHC: Primary healthcare
VA: Verbal autopsy
VAPAR: Verbal Autopsy with Participatory Action Research
YA: Youth ambassador

## References

[1] Prost A, Colbourn T, Seward N, Azad K, Coomarasamy A, Copas A (2013). Women’s groups practising participatory learning and action to improve maternal and newborn health in low-resource settings: a systematic review and meta-analysis. Lancet.

[2] Seward N, Neuman M, Colbourn T, Osrin D, Lewycka S, Azad K (2017). Effects of women’s groups practising participatory learning and action on preventive and care-seeking behaviours to reduce neonatal mortality: a meta-analysis of cluster-randomised trials. PLoS Med.

[3] Sondaal AEC, Tumbahanghe KM, Neupane R, Manandhar DSD, Costello A, Morrison J (2018). Sustainability of community-based women’s groups: reflections from a participatory intervention for newborn and maternal health in Nepal. Community Dev J..

[4] O’Meara WP, Tsofa B, Molyneux S, Goodman C, McKenzie FE (2011). Community and facility-level engagement in planning and budgeting for the government health sector—a district perspective from Kenya. Health Policy (New York).

[5] Doherty T, Oliphant N, Sanders D (2017). Community Health Workers delivering child health interventions: evidence-base and key considerations. World Nutr.

[6] World Health Organization. Report of the International Conference on Primary Health Care. International Conference on Primary Health Care. Alma Ata; 1978.

[7] United Nations (2015). Transforming our world: the 2030 Agenda for Sustainable Development.

[8] World Health Assembly. Strengthening integrated, people-centred health services. Resolution WHA69.24. Geneva; 2016.

[9] Frances F, La Parra D, Asunción M, Román M, Ortiz-Barreda G, Briones-Vozmediano E (2016). Toolkit on social participation.

[10] World Health Organization. Promoting participatory governance social participation and accountability. https://www.who.int/activities/promoting-participatory-governance-social-participation-and-accountability. Accessed 1 July 2020.

[11] Rifkin SB (2014). Examining the links between community participation and health outcomes: a review of the literature. Health Policy Plan..

[12] George AS, Mehra V, Scott K, Sriram V (2015). Community participation in health systems research: a systematic review assessing the state of research, the nature of interventions involved and the features of engagement with communities. PLoS ONE.

[13] South African Government (1996). The constitution of South Africa.

[14] Cleary SM, Molyneux S, Gilson L (2013). Resources, attitudes and culture: an understanding of the factors that influence the functioning of accountability mechanisms in primary health care settings. BMC Health Serv Res..

[15] Moosa S, Derese A, Peersman W (2017). Insights of health district managers on the implementation of primary health care outreach teams in Johannesburg, South Africa: a descriptive study with focus group discussions. Hum Resour Health..

[16] Schneider H, Besada D, Sanders D, Daviaud E, Rohde S (2018). Ward-based primary health care outreach teams in South Africa: developments, challenges and future directions. S Afr Heal Rev..

[17] South African National Department of Health (2018). Policy framework and strategy for ward-based primary healthcare outreach teams 2018/19–2023/24.

[18] Naidoo N, Railton J, Jobson G, Matlakala N, Marincowitz G, McIntyre JA (2018). Making ward-based outreach teams an effective component of human immunodeficiency virus programmes in South Africa. S Afr J HIV Med.

[19] White MS, Govender P, Lister HE (2017). Community health workers lensed through a South African backdrop of two peri-urban communities in KwaZulu-Natal. Afr J Disabil.

[20] Schneider H, Lehmann U (2016). From community health workers to community health systems: Time to widen the horizon?. Heal Syst Reform.

[21] Scott V, Gilson L (2017). Exploring how different modes of governance act across health system levels to influence primary healthcare facility managers’ use of information in decision-making: experience from Cape Town, South Africa Lucy Gilson. Int J Equity Health.

[22] Wickremasinghe D, Hashmi IE, Schellenberg J, Avan BI (2016). District decision-making for health in low-income settings: a systematic literature review. Health Policy Plan..

[23] Rifkin SB (2009). Lessons from community participation in health programmes: a review of the post Alma-Ata experience. Int Health.

[24] Mpumalanga Health Policy and Systems Research Learning Platform. https://www.vapar.org/.

[25] D’Ambruoso L, Van Der Merwe M, Wariri O, Byass P, Goosen G, Kahn K (2019). Rethinking collaboration: developing a learning platform to address under-five mortality in Mpumalanga province. S Afr Health Policy Plan.

[26] Hove J, D’Ambruoso L, Mabetha D, Van Der Merwe M, Byass P, Kahn K (2019). “Water is life”: Developing community participation for clean water in rural South Africa. BMJ Glob Heal.

[27] Oladeinde O, Mabetha D, Twine R, Hove J, Van Der Merwe M, Byass P (2020). Building cooperative learning to address alcohol and other drug abuse in Mpumalanga, South Africa: a participatory action research process. Glob Health Action..

[28] Witter S, Van der Merwe M, Twine R, Mabetha D, Hove J, Goosen G (2020). Verbal autopsy with participatory action research (VAPAR) programme in Mpumalanga, South Africa: protocol for evaluation. BMJ Open.

[29] Gilson L (ed). Health policy and systems research—a methodology reader. Alliance for Health Policy and Systems Research, World Health Organization. World Health Organization; 2012.

[30] Lincoln Y, Lynham S, GUba E. Chapter 6: Paradigmatic controversies, contradictions, and emerging confluences, revisited | University of Queensland. In 2013. p. 199–265.

[31] Pawson R, Tilley N. Realistic evaluation. London: Sage Publications; 1997.

[32] Jagosh J, Pluye P, Wong G, Cargo M, Salsberg J, Bush PL (2014). Critical reflections on realist review: insights from customizing the methodology to the needs of participatory research assessment. Res Synth Methods.

[33] Statistics South Africa. Mid-year population estimates 2019. Pretoria; 2019.

[34] Pillay-van Wyk V, Msemburi W, Laubscher R, Dorrington RE, Groenewald P, Glass T (2016). Mortality trends and differentials in South Africa from 1997 to 2012: second National Burden of Disease Study. Lancet Glob Heal.

[35] World Bank (2018). Overcoming poverty and inequality in South Africa—an assessment of drivers, constraints and opportunities overcoming poverty and inequality in South Africa.

[36] Statistics South Africa (2019). Sustainable development goals (SDGs) country report 2019—South Africa.

[37] World Health Organization (2014). Global status report on noncommunicable diseases 2014.

[38] Statistics South Africa (2016). Mortality and causes of death in South Africa, 2016: findings from death notification.

[39] Ataguba JE, Akazili J, McIntyre D (2011). Socioeconomic-related health inequality in South Africa: evidence from general household surveys. Int J Equity Health.

[40] McLaren Z, Ardington C, Leibbrandt M. Distance as a barrier to health care access in South Africa. A Southern Africa Labour and Development Research Unit Working Paper Number 97. Vol. 97, Working Paper Series. Cape Town: Southern Africa Labour and Development Research Unit, University of Cape Town; 2013.

[41] MRC/Wits-Agincourt Unit: Rural Public Health and Health Transitions Research Unit. https://www.agincourt.co.za/.

[42] Kahn K, Collinson MA, Xavier Gómez-olivé F, Mokoena O, Twine R, Mee P (2012). Profile: Agincourt health and socio-demographic surveillance system. Int J Epidemiol.

[43] Chang AY, Xavier Gómez-Olivé F, Payne C, Rohr JK, Manne-Goehler J, Wade AN (2019). Chronic multimorbidity among older adults in rural South Africa. BMJ Glob Health.

[44] Gómez-Olivé FX, Angotti N, Houle B, Klipstein-Grobusch K, Kabudula C, Menken J (2013). Prevalence of HIV among those 15 and older in rural South Africa. AIDS Care.

[45] Wariri O, D’Ambruoso L, Twine R, Ngobeni S, van der Merwe M, Spies B (2017). Initiating a participatory action research process in the Agincourt health and socio–demographic surveillance site. J Glob Health.

[46] D’Ambruoso L, Kahn K, Wagner RG, Twine R, Spies B, Van der Merwe M (2016). Erratum to: Moving from medical to health systems classifications of deaths: extending verbal autopsy to collect information on the circumstances of mortality. Glob Heal Res Policy..

[47] Ghaffar A, Langlois EV, Rasanathan K, Peterson S, Adedokun L, Tran NT. Strengthening health systems through embedded research. Bull World Health Organ. 2017;95(2):87. 10.2471/BLT.16.189126 PMC5327943 28250505

[48] Olivier J, Whyle E, Gilson L. Technical brief—embedded health policy and systems research. Alliance for Health Policy and Systems Research; 2018.

[49] Vindrola-Padros C, Pape T, Utley M, Fulop NJ (2017). The role of embedded research in quality improvement: a narrative review. BMJ Qual Saf.

[50] Langlois EV, Mancuso A, Elias V, Reveiz L (2019). Embedding implementation research to enhance health policy and systems: a multi-country analysis from ten settings in Latin America and the Caribbean. Health Res Policy Syst..

[51] Rajan D, Mathurapote N, Putthasri W, Posayanonda T, Pinprateep P, de Courcelles S (2019). Institutionalising participatory health governance: lessons from nine years of the National Health Assembly model in Thailand. BMJ Glob Health.

[52] Adeleye OA, Ofili AN (2010). Strengthening intersectoral collaboration for primary health care in developing countries: can the health sector play broader roles?. J Environ Public Health..

[53] Chircop A, Bassett R, Taylor E (2015). Evidence on how to practice intersectoral collaboration for health equity: a scoping review. Crit Public Health.

[54] Mahlangu P, Vearey J, Goudge J (2018). Multisectoral (in)action: towards effective mainstreaming of HIV in public sector departments in South Africa. Afr J AIDS Res.

[55] Tangcharoensathien V, Srisookwatana O, Pinprateep P, Posayanonda T, Patcharanarumol W (2017). Multisectoral actions for health: challenges and opportunities in complex policy environments. Int J Health Policy Manag.

[56] Morrison J, Akter K, Jennings HM, Kuddus A, Nahar T, King C (2019). Implementation and fidelity of a participatory learning and action cycle intervention to prevent and control type 2 diabetes in rural Bangladesh. Glob Health Res Policy.

[57] Orgill M, Nxumalo N, Amde W, Erasmus E, Lehmann U, Goudge J, et al. Health Policy and Systems Research: Needs, challenges and opportunities in South Africa-a university perspective. In: Padarath A, English R, editors. South African Health Review 2012/13. Durban: Health Systems Trust; 2013.

[58] van Ryneveld M, Whyle E, Brady L (2020). What is COVID-19 teaching us about community health systems? A reflection from a rapid community-led mutual aid response in Cape Town, South Africa. Int J Health Policy Manag..

[59] Rajan D, Koch K, Rohrer K, Bajnoczki C, Socha A, Voss M (2020). Governance of the Covid-19 response: a call for more inclusive and transparent decision-making. BMJ Glob Health.

